# Current neurologic treatment and emerging therapies in CDKL5 deficiency disorder

**DOI:** 10.1186/s11689-021-09384-z

**Published:** 2021-09-16

**Authors:** Heather E. Olson, Carolyn I. Daniels, Isabel Haviland, Lindsay C. Swanson, Caitlin A. Greene, Anne Marie M. Denny, Scott T. Demarest, Elia Pestana-Knight, Xiaoming Zhang, Ahsan N. Moosa, Andrea Fidell, Judith L. Weisenberg, Bernhard Suter, Cary Fu, Jeffrey L. Neul, Alan K. Percy, Eric D. Marsh, Timothy A. Benke, Annapurna Poduri

**Affiliations:** 1 Division of Epilepsy and Clinical Neurophysiology, Department of Neurology, Boston Children’s Hospital, 300 Longwood Avenue, Mailstop 3063, Boston, MA 02115 USA; 2 Division of Pediatric Neurology, University of Saskatchewan, Saskatoon, SK Canada; 3 Children’s Hospital Colorado, University of Colorado, School of Medicine, Aurora, CO USA; 4 Department of Pediatrics, School of Medicine, University of Colorado, Aurora, CO USA; 5 Epilepsy Center, Neurological Institute, Cleveland Clinic, Cleveland, OH USA; 6 Department of Pediatric Neurology, Washington University School of Medicine, St. Louis, MO USA; 7 Division of Child Neurology, Texas Children’s Hospital, Departments of Neurology and Pediatrics, Baylor College of Medicine, Houston, TX USA; 8 Department of Pediatrics, Vanderbilt University Medical Center, Nashville, TN USA; 9 Department of Pediatrics, University of Alabama at Birmingham, Birmingham, AL USA; 10 Division of Child Neurology, Children’s Hospital of Philadelphia, Departments of Neurology and Pediatrics, Perelman School of Medicine, University of Pennsylvania, Philadelphia, PA USA; 11 Departments of Pharmacology, Neurology, and Otolaryngology, School of Medicine, University of Colorado, Aurora, CO USA

**Keywords:** CDKL5 deficiency disorder, Developmental encephalopathy, Epileptic encephalopathy, Ketogenic diet, Vagus nerve stimulator, Movement disorders, Clinical trials, Emerging therapies

## Abstract

**Background:**

CDKL5 deficiency disorder (CDD) is associated with refractory infantile onset epilepsy, global developmental delay, and variable features that include sleep, behavioral disturbances, and movement disorders. Current treatment is primarily symptom-based and informed by experience in caring for this population.

**Methods:**

We describe medication and non-medication approaches to treatment of epilepsy and additional key neurologic symptoms (sleep disturbances, behavioral issues, movement disorders, and swallowing dysfunction) in a cohort of 177 individuals meeting criteria for CDD, 154 evaluated at 4 CDKL5 Centers of Excellence in the USA and 40 identified through the NIH Natural History Study of Rett and Related Disorders.

**Results:**

The four most frequently prescribed anti-seizure medications were broad spectrum, prescribed in over 50% of individuals. While the goal was not to ascertain efficacy, we obtained data from 86 individuals regarding response to treatment, with 2-week response achieved in 14–48% and sustained 3-month response in 5–36%, of those with known response. Additional treatments for seizures included cannabis derivatives, tried in over one-third of individuals, and clinical trial medications. In combination with pharmacological treatment, 50% of individuals were treated with ketogenic diet for attempted seizure control. Surgical approaches included vagus nerve stimulators, functional hemispherectomy, and corpus callosotomy, but numbers were too limited to assess response. Nearly one-third of individuals received pharmacologic treatment for sleep disturbances, 13% for behavioral dysregulation and movement disorders, and 43% had gastrostomy tubes.

**Conclusions:**

Treatment for neurologic features of CDD is currently symptom-based and empiric rather than CDD-specific, though clinical trials for CDD are emerging. Epilepsy in this population is highly refractory, and no specific anti-seizure medication was associated with improved seizure control. Ketogenic diet is commonly used in patients with CDD. While behavioral interventions are commonly instituted, information on the use of medications for sleep, behavioral management, and movement disorders is sparse and would benefit from further characterization and optimization of treatment approaches. The heterogeneity in treatment approaches highlights the need for systematic review and guidelines for CDD. Additional disease-specific and disease-modifying treatments are in development.

**Supplementary Information:**

The online version contains supplementary material available at 10.1186/s11689-021-09384-z.

## Background

CDKL5 deficiency disorder (CDD) is an X-linked disorder that represents one of the more common causes of genetic childhood-onset developmental and epileptic encephalopathy, with an estimated prevalence of one in 40,000 to 60,000 births [1–3]. CDD was previously considered an early onset seizure variant of Rett syndrome [2, 4], but has since been distinguished due to the disorder’s more severe developmental delays; increased likelihood of epilepsy, sleep abnormalities, and gastrointestinal problems; and lack of early regression following a period of normal development that is typical in Rett [5–10]. In 2019, minimum diagnostic criteria for CDD were proposed to include the presence of a pathogenic or likely pathogenic variant in *CDKL5*, epilepsy onset within 1 year of age, and motor and cognitive delays [2]. Key clinical features include refractory epilepsy, with onset at less than 3 months in 90% of individuals (median 5 weeks, IQR 3 to 8 weeks) [1], hypotonia, severe developmental delay/intellectual disability, and cortical visual impairment [1, 2, 10]. Both cognitive impairment and refractory epilepsy in individuals with CDD are particularly severe, and fewer than half of individuals have reported a period of seizure freedom of more than 2 months, with only 12% experiencing seizure freedom for more than 12 months [3, 10].

Patients with CDD typically have several seizure types, with one study reporting an average of 2.8 (SD 1.4) different seizure types at any time [2]. Epileptic spasms (with or without hypsarrhythmia), tonic seizures, and generalized tonic-clonic seizures are most common, and seizures with multiple phases (such as the hypermotor-tonic-spasms sequence) are also common [1, 2]. Epilepsy tends to evolve to generalized or mixed focal and generalized patterns [2].

Data on response to available treatment for epilepsy in CDD remain limited, though case series suggest that symptoms are highly refractory to treatment [1–3, 6, 11–15]. One retrospective review of anti-seizure medication (ASM) response in 39 individuals with CDD demonstrated a responder rate (> 50% reduction in seizure frequency) to at least one ASM or to ketogenic diet (KD) of 69% at 3 months, falling to 24% at 12 months [11]. Additionally, 31% of individuals experienced seizure aggravation with at least one ASM [11]. Data from the International CDKL5 Disorder Database (ICDD) further suggest that the most commonly prescribed ASMs are broad-spectrum medications including clobazam, topiramate, and levetiracetam, and that almost one-third of individuals are treated with steroids at some point [3]. Limited retrospective data suggest that KD and vagus nerve stimulator (VNS) treatment may have some benefit [12, 13].

Although seizures are an early and prominent clinical feature of CDD, other neurological symptoms are equally prominent, including sleep dysfunction, behavioral dysregulation, movement disorders, and swallowing dysfunction. The majority of individuals with CDD experience clinically significant sleep problems [7, 16–18], with concerns including diurnal problems, bruxism, night screaming, night laughing, and night waking [7]. Comorbidities of CDD such as severe hypotonia and autonomic dysfunction may contribute to obstructive airway symptoms and central sleep apnea, respectively [16]. Reports on behavioral dysregulation associated with CDD are limited; however, there are several case reports of individuals with autistic features and perseverative, self-centered behavior [5, 15, 19–24]. Movement disorders described in individuals with CDD include dyskinesias, chorea, ballismus, myoclonus, and hand stereotypies [6, 16, 24]. Dysphagia is common in CDD and data from the International CDKL5 Disorder Database suggested that 20.7% of individuals were fed exclusively by gastrostomy or nasogastric tube [7]. Like epilepsy, these additional neurological symptoms negatively impact individuals' quality of life and may be treatable by medication and non-medication approaches, thus are important to understand how to manage [18, 25].

We aim to describe current treatment approaches and emerging therapies for epilepsy and additional treatable neurological symptoms (sleep dysfunction, behavioral dysregulation, movement disorders, and swallowing dysfunction) in individuals with CDKL5 deficiency disorder at experienced centers. This complements ongoing efforts towards defining treatment guidelines for CDD.

## Methods

### CDD cohort

In collaboration with the International Foundation for CDKL5 Research, dedicated Centers of Excellence (COEs) were established for expert care of children with CDD and parallel clinical research [1]. Our cohorts consisted of individuals with CDD seen in COEs at Boston Children’s Hospital (BCH, *n* = 56), Cleveland Clinic Foundation (CCF, *n* = 30), University of Colorado/Children’s Hospital Colorado (CHCO, *n* = 51), and Baylor College of Medicine (BCM, *n* = 17). BCM provided data exclusively on movement disorders in CDD patients. In partnership with the NIH-funded Natural History Study of Rett and Related Disorders (NHS, U54 HD061222; NCT02738281), data related to anti-seizure treatments, as well as medications for sleep dysfunction and behavioral dysregulation, were gathered from an additional cohort of 40 individuals with CDD that represents individuals enrolled across 10 clinical sites, and excluded individuals already captured in BCH, CCF, and CHCO COE cohorts. Inclusion criteria included meeting consensus criteria for CDD: a pathogenic or likely pathogenic variant in *CDKL5* gene, motor and cognitive developmental delays, and epilepsy with onset in the first year of life [2]. Each center has an Institutional Review Board (IRB)-approved protocol for prospective and retrospective collection of clinical data, including data from medical records, on individuals with CDD. Clinical and genetic data are gathered using standardized clinical research forms across the Centers of Excellence, and maintained in separate REDCap databases. IRB approval exists for all sites in the NHS.

### Evaluation of neurologic management

Data were collected both retrospectively and prospectively through the dedicated COEs as part of a clinic-based research study. Physicians in the Centers of Excellence included neurologists and other pediatric specialists, for example gastroenterologists. Medical records and standardized clinical research forms were reviewed to evaluate medication and non-medication treatment approaches for epilepsy, sleep dysfunction, behavioral dysregulation, and movement disorders. For epilepsy, we tabulated ASMs used including response and tolerance, as well as use of ketogenic diet, vagus nerve stimulator, and epilepsy surgery. For ASMs, a positive response was defined as a 50% or greater reduction in at least one seizure type sustained for two time periods: 2 weeks and 3 months, as best estimated based on retrospective review of medical records including clinic, hospital, and phone notes. NHS data were obtained as part of initial and yearly research visits. Data on sleep and behavioral medications were collected at BCH, CCF, and through NHS. Data on movement medications were collected at BCH, CCF, and BCM. Data on gastrostomy tubes were collected from BCH and CHCO COEs. Data on response to treatment were not part of clinical research forms or NHS data collection and were only collected at BCH and CCF based on review of medical records. Though EEG data were available for some patients, EEG data were not used routinely to quantify response to treatment. Cannabis derivatives, including Epidiolex and non-FDA approved derivatives, are described separately because cannabis derivatives were included as a general category, without specifying whether they referred to Epidiolex or not, in some of the dataset. Seizure data were gathered for COE patients based on medical record review and from standardized research forms. All seizure types occurring in CDD, including infantile spasms, were included. Seizure classification was conducted using International League Against Epilepsy (ILAE) guidelines [26].

### Statistical analysis

Descriptive statistical methods were used, using Microsoft Excel (Version 14.7.7) and IBM SPSS Statistics (Version 27).

## Results

### Demographic characteristics

Of 177 individuals with CDD seen at three CDKL5 COEs and 10 NHS sites across the USA, 82% of individuals were female (*n* = 145) and 18% were male (*n* = 32). *CDKL5* variants included truncating variants in 63, missense variants in the kinase domain in 44, partial or full-gene deletions in 20, splice variants in 6, and unknown protein impacts in 4. All variants were de novo when inheritance information was available. Median age at time of data collection was 8.1 years (range 8 months to 39 years). Data on race and ethnicity were available for 126 individuals, with 82% of individuals reported as white, 6% reported as Asian, and 3% reported as Black or African American. In addition, 4% reported membership in more than one racial category, 0.7% reported racial category as “other,” 0.7% did not provide racial category, and for 1%, racial category was unknown. In addition to racial category, 12% of individuals reported Hispanic, Latino/a or Spanish origin.

### Seizure types

Seizure types were known for 136 patients from three COEs. The most common seizure types at any time point included spasms (*n* = 90, 66%), generalized tonic (*n* = 82, 60%), focal seizures (*n* = 69, 51%), generalized myoclonic (*n* = 60, 44%), and generalized tonic-clonic (*n* = 57, 42%). Seizures with multiple phases were seen in 41% of patients.

### Anti-seizure medications

Data on ASM treatment were available from 168 individuals from the three COEs and through NHS. The median number of anti-seizure medications prescribed per individual (lifetime use) was 6 (range 0 to 18). In total, 33 different ASMs were prescribed in this group of individuals. The most frequently prescribed medications (prescribed in more than 50% of individuals) were levetiracetam (*n* = 136), topiramate (*n* = 107), clobazam (*n* = 100), and phenobarbital (*n* = 89). For 86 individuals with treatment response data for the most frequently prescribed medications, 2-week sustained response, defined as 50% or greater reduction in at least one seizure type during this time frame, was reported for 14 to 48% of individuals (of those with known response) (Table 1). A 3-month response, however, was reported in only 5–36%. Worsening seizures attributable to ASMs were reported in 39 individuals (39/88, 44%). Details on ASM response for all medications prescribed can be found in Table 1.

**Table 1:** Summary of response of 86 individuals with CDD to anti-seizure medications

Medication	# individuals	# individuals with known response	2-week response (***n***, (% of those prescribed the medication with known response))	3-month response (***n***, (% of those prescribed the medication with known response))	Worsening seizures (***n***, (% of those prescribed the medication with known response))	No change (***n***, (% of those prescribed the medication with known response))
Levetiracetam	75	56 (75%)	8 (14%)	3 (5%)	5 (9%)	43 (77%)
Topiramate	57	38 (67%)	10 (26%)	5 (13%)	2 (5%)	26 (68%)
Phenobarbital	53	35 (66%)	13 (37%)	3 (9%)	1 (3%)	21 (60%)
Clobazam	52	25 (48%)	12 (48%)	9 (36%)	4 (16%)	10 (40%)
Vigabatrin	41	27 (66%)	15 (56%)	9 (33%)	2 (7%)	9 (33%)
Valproic acid	40	25 (63%)	9 (36%)	7 (28%)	0	16 (64%)
Oxcarbazepine	31	20 (65%)	5 (25%)	2 (10%)	3 (15%)	12 (60%
ACTH	30	19 (19%)	8 (42%)	0	1 (5%)	10 (53%
Clonazepam	30	11 (37%)	0	0	2 (18%)	9 (82%)
Prednisolone/prednisone	28	21 (75%)	7 (33%)	0	1 (5%)	13 (62%)
Rufinamide	28	15 (54%)	7 (47%)	4 (27%)	3 (20%)	6 (40%)
Lamotrigine	26	15 (58%)	6 (40%)	2 (13%)	1 (7%)	8 (53%)
Zonisamide	22	13 (59%)	0	0	1 (8%)	12 (92%)
Cannabidiol (Epidiolex)	19	14 (74%)	4 (29%)	3 (21%)	4 (29%)	6 (43%)
Lacosamide	12	9 (75%)	0	0	2 (22%)	7 (78%)
Felbamate	11	5 (45%)	1 (20%)	1 (20%)	0	4 (80%)
Carbamazepine	10	6 (60%)	0	0	0	6 (100%)
Phenytoin	10	8 (80%)	1 (13%)	1 (13%)	2 (25%)	5 (63%)
Clorazepate	7	4 (57%)	0	0	1 (25%)	3 (75%)
Gabapentin	5	4 (80%)	0	0	0	4 (100%)
Perampanel	5	4 (80%)	0	0	0	4 (100%)
Ethosuximide	4	1 (25%)	0	0	0	1 (100%)
Diazepam	3	1 (33%)	0	0	0	1 (100%)
Lorazepam	3	2 (67%)	1 (50%)	1 (50%)	2 (100%)	0
Nitrazepam	3	1 (33%)	0	0	0	1 (100%)
Pregabalin	3	1 (33%)	0	0	0	1 (100%)
Methsuximide	2	1 (50%)	0	0	0	1 (100%)
Midazolam	2	2 (100%)	0	0	0	2 (100%)
Acetazolamide	1	0	0	0	0	0
Brivaracetam	1	1 (100%)	1 (100%)	1 (100%)	0	0
Fosphenytoin	1	1 (100%)	0	0	0	1 (100%)
Stiripentol	1	0	0	0	0	0
Tiagabine	1	1 (100%)	0	0	0	1 (100%)

Sixty-one individuals (*n* = 61 of 168, 36%), out of the total number of individuals with ASM data, were treated with cannabis derivatives, including FDA-approved cannabidiol (Epidiolex), and non-FDA-approved cannabis derivatives. This was predominantly in combination with other epilepsy treatments. Of 86 individuals for whom Epidiolex was differentiated from non-FDA-approved cannabis derivatives, 19 reported using Epidiolex (22%) and 18 reported using non-FDA-approved cannabis derivatives. Two-week response, for those with known response, was reported for Epidiolex in 29% of individuals and sustained for 3 months in 21%, compared to 50% of those with known response for both time periods for non-FDA-approved cannabis derivatives (Table 2).

**Table 2:** Summary of response to cannabis derivatives for individuals with CDD from two COEs

Treatment	# individuals	# individuals with known response	2-week response (***n***, (% of those taking with known response))	3-month response (***n***, (% of those taking with known response))	Worsening seizures (***n***, (% of those taking with known response))	No change (***n***, (% of those taking with known response))
Cannabidiol (Epidiolex)	19	14 (74%)	4 (29%)	3 (21%)	4 (29%)	6 (43%)
Non-FDA-approved cannabis derivative	18	4 (19%)	2 (50%)	2 (50%)	1 (25%)	2 (50%)

Medications and treatments reported to be most helpful for seizure control, based on a specific question on our standardized research forms as well as on caregiver and clinician’s impression, were listed for 41 individuals and included the following (percentages are based on number of patients who were prescribed each medication): clobazam (*n* = 10 of 28, 36%), ketogenic diet (*n* = 6 of 28, 21%), topiramate (*n* = 5 of 29, 17%), prednisolone/prednisone (*n* = 5 of 15, 33%), ACTH (*n* = 5 of 17, 29%), valproic acid (*n* = 4 of 22, 18%), lamotrigine (*n* = 4 of 12, 33%), vigabatrin (*n* = 3 of 22, 14%), rufinamide (*n* = 3 of 17, 18%), Epidiolex (*n* = 2 of 7, 29%), clonazepam (*n* = 2 of 15, 13%), non-FDA-approved cannabis derivatives (*n* = 2 of 4, 50%), zonisamide (*n* = 1 of 12, 8%), brivaracetam (*n* = 1 of 1, 100%), lacosamide (*n* = 1 of 7, 14%), felbamate (*n* = 1 of 6, 17%), and levetiracetam (*n* = 1 of 37, 3%).

### Ketogenic diet

Eighty-nine of 177 individuals (50%) were placed on the ketogenic diet at some point in their treatment course. Details on the use of KD were available for 54 patients. Median age of diet initiation was 1.92 years (range 6 weeks to 10 years), with 13 (24%) starting before 12 months. Thirty-two individuals used the diet for a finite period of time (median of 11 months, range 1 month to 8 years), and 15 individuals remained on the diet at last clinic visit. Duration of use was unknown for the remaining individuals (*n* = 7). Seizure frequency at the time of diet initiation was daily in 42 of 54 individuals (78%, ranging from 1 to 30 seizures per day), weekly for one individual, and unknown for the remaining 11 individuals. Six of the 54 total individuals who tried KD (11%) reported increase in seizures on the diet, 2 of whom had increased seizures at higher ratios but improvement in seizures at lower ratios. Most common side effects and complications reported, consistent with expectations for KD treatment, were gastrointestinal issues including reflux, constipation, vomiting, and gastrointestinal distress (*n* = 7), acidosis (*n* = 6), feeding difficulties (*n* = 5), hypoglycemia (*n* = 4), lethargy (*n* = 3), and aspiration pneumonia (*n* = 2).

Clinically significant seizure response to the diet was known for 44 individuals. Twenty-two of 44 individuals (50%) were responders (time point of evaluation unspecified). In three individuals (considered as non-responders), benefit was more modest, with a decrease in seizure frequency below 50%. Twenty-two individuals (50%) did not respond to the diet. Although the mean age of those who responded to KD (1.75 years) was lower than that of non-responders (3.0 years), this difference was not statistically significant with Mann-Whitney *U* test (*p* = 0.105). Eight of 10 individuals (80%) who started the diet at < 12 months had a clinically significant reduction in seizures, and two individuals (20%) did not respond to KD treatment. Thirteen of 31 individuals who started the diet at or after age 1 year (of those with known response) responded to the diet (42%). A Fisher’s exact test comparing response between individuals who started KD at < 12 months vs ≥ 12 months was statistically significant (*p* = 0.067). Seven of 22 previously noted responders reported subjective cognitive improvements based on caretaker reports associated with use of the diet, including improved social interaction and mood, increased alertness, and improved developmental progress. Cognitive benefits were reported in four non-responders. Nineteen individuals did not have cognitive improvements with ketogenic diet, and information was not available for 5 individuals. Summary of response to KD, of those with known response, can be found in Table 3. Additional details are included in Supplemental Table 1 (see Supplemental Tables).

**Table 3:** Summary of response of 44 individuals with known response to ketogenic diet

	Age at start of diet (years, median/mean)	Cognitive improvements reported	Side effects reported
**Responders, 50% (*n*= 22)**	1.17/1.75	32% (*n* = 7)	64% (*n* = 14)
**Non-responders, 50% (*n*= 22)**	1.92/3.00	18% (*n* = 4)	27% (*n* = 6)

### VNS and epilepsy surgery

Thirty-six of 177 individuals (20%) reported treatment with a vagus nerve stimulator. Median age at VNS placement was 5 years (range 1 year 9 months to 13 years) for 16 individuals with detailed information. Seizure frequency at VNS placement was daily for 12 individuals, weekly for one individual, and unknown for three individuals. Seven individuals (7/16, 43%) reported a decrease in seizure frequency post-implantation at any time point, three individuals reported a decrease in seizure intensity (3/16, 18%), and two individuals reported a decrease in seizure duration (2/16, 12%). There was not enough information in medical records to assess percentage of seizure decrease. Reported side effects included swallowing difficulties in one individual and initial increase in frequency or severity of seizures in two individuals which subsequently improved while continuing with VNS treatment. Additional details on response to VNS treatment can be found in Table 4.

**Table 4:** Response of 16 individuals with CDD to vagus nerve stimulator

Subject ID	Age at placement	Seizure frequency at start of VNS	Seizure response	Side effects
A11	Unknown	Daily	No change in seizures	None reported
A20	9 years 4 months	2–3/day	Decreased frequency and severity, decreased duration with magnet	Initial increase in frequency of seizures prior to adjustment of settings
A21	6 years 3 months	5–6/day	Decreased severity	None reported
A24	1 year 9 months	Daily	Decreased frequency and severity	Swallowing difficulties
A25	4 years 3 months	Daily	Minimal effect	None reported
A26	4 years	Unknown	Decreased frequency	None reported
A28	3 years 2 months	Daily	Decreased frequency	Initial increase in severity of seizures
A31	2 years 8 months	8–10 clusters/day	Decreased frequency, response to magnet	None reported
A35	10 years	Weekly	Decreased duration	None reported
A79	13 years	Unknown	Decreased duration	None reported
B7	7.5 years	Daily	No change in seizures	None reported
B8	5 years	Daily	No change in seizures	None reported
B11	Unknown	Daily	No change in seizures	None reported
B14	Unknown	Daily	Unknown	Unknown
B15	2 years 3 months	Daily	Slight reduction in seizures	None reported
B22	7 years	10–14/day	Reduced seizures to 5–6/day	None reported

Of 137 individuals, 6 underwent other surgical treatments for epilepsy. One individual had a right functional hemispherectomy at 2 years of age, and seizures persisted post-surgery. Five individuals reported undergoing corpus callosotomy for seizure control, and data on response were available for three of them. One individual reported a significant reduction in seizures, with recurrence after an unspecified period of time. Another individual reported reduction in duration of one seizure type (tonic seizures), and a repeat corpus callosotomy is being considered. Another individual had complete resolution of atonic seizures for approximately 1 year, followed by recurrence at a lower frequency.

### Other neurologic symptoms

Sleep disturbances were reported at any time point in 61 of 86 individuals (71%). Sleep medications were used in 40 of 126 individuals (32%), with a range of 1–5 sleep medications used per person (median = 1). The most commonly prescribed sleep medication was melatonin (*n* = 30), with 10 individuals reporting an improvement in sleep with this medication (30%). Sixteen of 127 individuals (13%) received medications for behavioral management including selective serotonin or serotonin and norepinephrine reuptake inhibitors, atypical antipsychotics, alpha-2 adrenergic receptor agonists, benzodiazepines, and stimulants, the most common indication being irritability (*n* = 9). Additional details on response to prescribed sleep and behavioral medications can be found in Supplemental Table 2 (see Supplemental Tables). Thirty-five of 154 individuals from four COEs (23%) were described to have movement disorders, the most common being choreoathetosis (*n* = 16), dystonia (*n* = 7), and dyskinesia (*n* = 4). Of 24 individuals with information on pharmacological treatment, three individuals received medications for choreoathetosis (clonidine, tetrabenazine, and risperidone) (13%). Additionally, at least 40 patients (43.5% of 92 with available data) had a gastrostomy tube for feeding difficulties including dysphagia. Of 24 patients with data on modified barium swallow studies, dysphagia with risk of aspiration was noted in 14 (58.3%).

## Discussion

In this assessment of current treatment approaches for neurologic aspects of CDD at academic medical centers throughout the USA, we demonstrate that epilepsy is highly refractory, that the most common ASMs prescribed are broad spectrum as appropriate for generalized or mixed-type epilepsy with both focal and generalized seizure types, and that response rate to KD is likely better than to medication but still variable. Data on VNS and other surgical approaches for epilepsy are limited to our report of exposures, as are data on treatment of other neurologic symptoms, including sleep, behavioral, and movement disorders. Disease-specific clinical trials are underway, and emerging therapies are reviewed below.

The individuals described with CDD were treated for multiple generalized and focal seizure types including tonic, tonic-clonic, myoclonic, and spasms, as well as seizures with multiple phases. A separate study evaluating response of epileptic spasms to first-line treatments in CDD is underway [27]. Response rates to ASMs in our cohort were quite low, ranging from 5 to 36% at 3 months for the most prescribed medications. It is important to note that patients received multiple ASMs simultaneously, as well as other treatments including cannabis derivatives, trial drugs, KD, and epilepsy surgery, which makes it difficult to draw conclusions about the efficacy of any individual treatment. Clinician and caregiver impression of the most helpful epilepsy treatments included clobazam, topiramate, steroids, valproic acid, and lamotrigine. Though we cannot make firm conclusions about efficacy, this is consistent with one additional report in the literature, also based on retrospective review of caretaker and clinician impression, stating that medications with the highest rates of seizure reduction at 3 months include vigabatrin, clobazam, valproic acid, steroids, lamotrigine, felbamate, and zonisamide [11]. The range of response rates to each ASM in this report was broad, with a maximum of 33% [11]. Despite a low response rate at 3 months, these medications continue to be the most frequently prescribed. While one publication suggested that carbamazepine may exacerbate seizures in CDD, a more recent retrospective study based on a cohort of 21 individuals with CDD reported that sodium channel blockers (SCBs) led to a greater than 50% seizure reduction for more than 6 months in 31.6% of participants, and to seizure freedom for more than 5 years in 15.8%, proposing SCBs as a potentially beneficial therapy in well-selected individuals with CDD-related epilepsy [11, 28]. SCBs were used in our cohort, but we did not control for evaluation of individual treatments as was done in Aledo-Serrano’s study, so we cannot draw conclusions on the efficacy of particular ASMs by pharmacologic category. Another recent study based on a group of 14 individuals with CDD found that 5 participants responded to a combination of vigabatrin and zonisamide for epileptic spasms [29]. However, our methodology did not allow us to evaluate specific combinations of ASMs and their effects on seizure control. Forty-four percent of the individuals in our study reported worsening seizures associated with ASM treatment, similar to 31% with seizure aggravation in a prior study, but this exacerbation may be due to the evolution of the disease rather than to medications [11]. None of the patients in our cohort had worsening with carbamazepine, and we did not observe a higher rate of seizure exacerbation with use of SCBs compared to other ASMs, in contrast to the one previous study showing seizure exacerbation with carbamazepine [11].

Our response rate to cannabis derivatives, 21–50% at 3 months, is in line with what is reported in the only study in the literature that reports the effect of Epidiolex on seizures in individuals with CDD [30], where 41% were reported as responders at 3 months. Results of this prospective open-label interventional study showed that individuals with CDD had a reduction in motor seizures from median 66.4 per 28 days to 35.8 at 12 weeks, with stable frequency at 48 weeks [30]. The validity of our results is limited due to missing data for those who used non-FDA approved products.

Ketogenic diet is a common treatment for refractory epilepsy [31]. Compared to the prior report on use of KD in CDD reporting a median age of initiation of 4.8 years, individuals in our cohort had a median age of initiation of 1.92 years [12]. Rate of improvement of seizures and cognition was similar compared to this prior study, but our study suggests a trend toward a higher percentage of individuals with response in those who initiated the diet before 1 year of age (80%) compared to those initiating diet after 1 year of age (42%) [12]. The difference did not reach statistical significance and would require further research to determine if efficacy may be higher with initiation in infancy.

Surgical approaches for refractory epilepsy in this series included VNS, functional hemispherectomy, and corpus callosotomy. In our cohort, 43% of individuals reported a decrease in seizure frequency and 18% reported a decrease in seizure intensity post-VNS implantation, compared to 68% and 60%, respectively, in a prior study [13]. Additionally, only 12% of our cohort reported a decrease in seizure duration, versus 72% of individuals from ICDD [13]. The lower rate of reported response in our study may be due to the comparatively small number of individuals who reported VNS use (*n* = 16), which may limit the representativeness of our sample. Additionally, the COEs represent quaternary care centers and may be biased towards a more severely affected group of individuals. Further study is required to assess corpus callosotomy and other surgical approaches to epilepsy treatment in CDD.

In addition to treatment for seizure control, nearly one-third of individuals received treatment for sleep disturbances, and 13% received treatment for behavioral dysregulation and movement disorders. Although the prevalence and characteristics of sleep, behavioral, and movement disorders in CDKL5 have been described in the literature [2, 7, 16, 17], data on treatment are limited. Further study is required in each of these areas to assess efficacy of treatments based on specific sub-types of symptoms.

At the 2019 Patient-Focused Drug Development Meeting for CDD, caregivers identified the top 3 most burdensome symptoms for CDD patients as global developmental delay (79%), epilepsy (63%), and gastrointestinal and feeding problems (48%) [32]. Behavioral disturbances were rated as most burdensome by 17% of caregivers, and sleep problems by 6%, while movement disorders were not selected by any respondents [32]. Additionally, a recent study reported that functional impairment, including lack of ability to sit, use hands, and communicate, was reported to have the greatest adverse impact on quality of life for individuals with CDD [25]. We focused on epilepsy and other neurologic aspects of CDD that are currently treatable in a clinical setting, but further studies characterizing therapy and educational approaches to improving functional impairment and global developmental delay in CDD would be beneficial.

### Review of emerging therapies

Over the last several years, CDD clinicians, researchers and pharmaceutical company partners have collaborated to bring a number of investigational drugs to clinical trials as well as explore emerging precision therapies for CDD. As of December 2020, there have been five clinical trials for the CDD population with seizure management as the primary outcome measure.

The neurosteroid ganaxolone (an analog of allopregnanolone), which is an allosteric modulator of GABA-A receptors, recently completed a phase III clinical trial (NCT03572933) that included a 17-week double blind phase [33, 34]. Results included a significant reduction in major motor seizure frequency in comparison with placebo (32.2% vs. 4.0%) [35], and an expanded access program is being developed for ganaxolone in CDD [36].

A phase II open-label study with soticlestat (OV935/TAK-935) was recently completed for individuals with CDD (NCT03694275) [37]. Soticlestat is a selective cholesterol 24-hydroxylase (CH24H) inhibitor that converts brain cholesterol to 24S-hydroxycholesterol (24HC), which is a positive allosteric modulator of the NMDA receptor [37, 38]. The results from the phase II study show that treated individuals with CDD (*n* = 11) experienced a 24% median reduction in major motor seizures during the 12-week maintenance period [37]. Five individuals with CDD who continued into the open label extension portion of the study achieved a 50% median motor seizure frequency reduction [37]. The Clinical Global Impression of Change as a secondary end point also demonstrated improvements [37].

Two investigator-initiated studies for therapeutics in CDD were an open-label fenfluramine trial (NCT03861871), and a phase II randomized, placebo-controlled crossover study of ataluren for the treatment of individuals with CDD with nonsense variants in *CDKL5* (NCT02758626). Ataluren, a non-aminoglycoside drug, targets pathogenic nonsense variants in other genetic diseases [39]. Results of recent in vitro studies of aminoglycoside drugs demonstrate that nonsense CDKL5 variants are efficiently suppressed, raising the question of utility of ataluren for CDD [39]. Fenfluramine acts on multiple receptors and is involved in the modulation of glutamate as well as increasing serotonin release and inhibiting reuptake [40]. Fenfluramine showed 90% reduction of generalized tonic clonic seizures in this small open label study, while ataluren did not show efficacy [41, 42].

To date, no disease-modifying therapy exists for CDD but several are in development. A protein transduction domain (TAT)-CDKL5 fusion protein was efficiently internalized by target cells and retained CDKL5 activity, suggesting that CDKL5 protein therapy may be an effective clinical tool for the treatment of CDD [43]. A splicing correction strategy has been applied to *CDKL5*, developing engineered U1snRNA variants that were able to rescue *CDKL5* mRNA splicing, protein synthesis, and function [44]. Other researchers induced escape of *CDKL5* from X chromosome inactivation by editing DNA methylation on the promoter of CDKL5 using a dCas9-TET1 fusion protein [45]. Recently, the first proof-of-concept study on gene replacement as a potential approach for the treatment of CDD was published, using adeno-associated virus (AAV)-*CDKL5* vectors in in vivo and in vitro models of CDD [46]. The authors cloned and produced AAV vectors expressing the major *CDKL5* brain isoforms and delivered them in a *Cdkl5* KO mouse model as well as in a *CDKL5*-mutant iPSC-derived neuron model [46]. Treatment in the mouse model led to improvements in motor functions and autistic-like behaviors compared to controls. In the neuron model, treatment led to an increased density of synaptic puncta and ameliorated the calcium signaling defect compared to controls [46]. Future proof-of-concept studies are required to answer fundamental questions [47], but this study represents an important beginning for demonstrating the utility of virus-mediated gene transfer in CDD [46].

### Limitations

This study is limited by the mixed prospective and retrospective study design. Individuals were seen a variable number of times at the centers and did not necessarily receive their primary management at these centers. Treatment response was exclusively obtained retrospectively from medical records, making any conclusion about efficacy quite limited, so we mainly demonstrate treatment patterns. We are also not able to comment on order of use or specific combinations of medications from this dataset. Historical data for individuals enrolled in NHS were gathered primarily from a baseline visit, with additional data coming from 61% of individuals (*n* = 25) seen for one follow-up, and 37% (*n* = 15) seen for two follow-ups. Lastly, the majority of our cohort was white and non-Hispanic, likely due to disparities in testing or referrals. Future research would benefit from including a broader and more diverse sample.

## Conclusion

Our study provides an overview of current treatment patterns for neurologic symptoms in CDD, including aspects of the disorder for which there is limited information in the literature such as treatment for sleep, behavior and movement disorders, as well as non-pharmacological treatments for epilepsy including ketogenic diet and surgery. By emphasizing the refractory nature of epilepsy in CDD and the lack of sustained effects of current treatments, we stress that new approaches are necessary to better care for this group of individuals. We provide a review of the latest emerging therapies for CDD, including clinical trial medications and novel treatment approaches under development. Our data complement efforts toward defining treatment guidelines for CDD, with the hope of adding disease-modifying therapies to the treatment arsenal in the near future.

## Supplementary Information

**Additional file 1: Supplemental Table 1.** Treatment response in 54 individuals with CDD treated with ketogenic diet. **Supplemental Table 2.** Response of 126 individuals with CDD to common sleep and behavioral medications

## Data Availability

The datasets generated and/or analyzed during the current study are not publicly available due to identifiable information, but de-identified data from the CDKL5 Centers of Excellence are available from the corresponding author upon reasonable request. Data stored in the NHS database are governed by an NIH acceptable data sharing agreement and are now available via the database of Genotypes and Phenotypes (dbGaP).

## Abbreviations

CDD: CDKL5 deficiency disorder
ASM: Anti-seizure medication
KD: Ketogenic diet
VNS: Vagus nerve stimulator
BCH: Boston Children’s Hospital
CCF: Cleveland Clinic Foundation
CHCO: University of Colorado/Children’s Hospital Colorado
NHS: Natural History Study
COE: Center of Excellence
IRB: Institutional Review Board
ILAE: International League Against Epilepsy
