# Supplementary material for: Current neurologic treatment and emerging therapies in CDKL5 deficiency disorder
Source: J Neurodev Disord. 2021 Sep 16;13:40. doi: 10.1186/s11689-021-09384-z (PMC8447578; doi:10.1186/s11689-021-09384-z)
Supplement: Supplementary file 1 — Additional file 1: Supplemental Table 1. Treatment response in 54 individuals with CDD treated with ketogenic diet. Supplemental Table 2. Response of 126 individuals with CDD to common sleep and behavioral medications [file 11689_2021_9384_MOESM1_ESM.docx]

**Supplemental Tables:**

Supplemental Table 1. Treatment response in 54 individuals with CDD treated with ketogenic diet

| **Subject ID** | **Age at start of diet** | **Seizure frequency at start of diet** | **Duration of diet** | **~50% or greater reduction in frequency of at least 1 seizure type?** | **Additional notes on seizure response** | **Cognitive improvements** | **Side effects/complications** |
| --- | --- | --- | --- | --- | --- | --- | --- |
| A2 | 2 years 11 months | 0-10/day | 1 year 9 months | Yes | Essentially seizure free, recurred after about 12 months | Improved social interaction | Hypoglycemia, acidosis |
| A3 | 1 year, 1 year 4 months | 1-10 clusters/day | Less than 3 days on each occasion | No | Unable to assess, discontinued due to intolerance | None reported | Vomiting, acidosis, hypoglycemia, decreased oral intake |
| A5 | 10 months | Daily | 8 months | Yes | Recurred to baseline with time | None reported | Acidosis, aspiration pneumonia, frequent illness |
| A7 | 5 months | 2-4/day | At least 8 months | Yes | Recurred to baseline after about 5 months | None reported | Transient hypoglycemia and acidosis, increased frequency of seizures at high ratio |
| A12 | 7 months | 3-5/day | 1 year 8 months | Yes | Recurred to baseline after about 7 months | None reported | Hypercalcemia, chronic *Clostridium difficile* infection |
| A13 | 3 years 9 months | 2-3/day | At least 11 months | No | No change in seizures | Improved alertness and muscle tone | Reflux |
| A15 | 5 years 9 months | 1-7/day | 4-6 months | No | No reduction, possible increase | None reported | Aspiration pneumonia |
| A20 | 5 years | 2-3/day | 5 years | Yes | Decrease in severity | Increased alertness, improved social interaction | Acidosis, lethargy, hypoglycemia, kidney stones |
| A21 | 2 years 6 months | 3-5 clusters/day | 2.5 years | Yes | Effects waned over time | Increased alertness, attention span, participation, improved eye contact and mobility | Seizures increased at high ratio |
| A24 | 1 year 5 months | Daily | 6 months | No | No change in seizures | None reported | None reported |
| A25 | 6 years | Unknown | 8 months | No | No change in seizures | None reported | None reported |
| A26 | 10 years | Daily | 2 years | No | Minimal change in seizures | None reported | Weight loss |
| A27 | 2 years 1 month | Daily | 6 months | Yes | Decrease in severity | Improved developmental progress and alertness | Feeding issues, constipation, reflux |
| A28 | 4 years 7 months | 3-4/day | 4.5 years | Yes | Decrease in severity | Increased alertness, improved social interaction | Reflux, GI distress, seizures increased at high ratio |
| A29 | 2 years 6 months | 2-3/day | At least 6 months | No | No change in seizures | None reported | None reported |
| A31 | 1 year 2 months | Daily | 6 months | No | Decreased frequency 25-50%, decreased severity | Increased alertness, improved mood | None reported |
| A33 | 3 years 10 months | 1/day | 3 months | No | No change in seizures | None reported | None reported |
| A34 | 2 years | Daily | 1 year | Yes | Decreased severity, eventual recurrence | None reported | None reported |
| A35 | Unknown, tried on 3 occasions | Unknown | Unknown | Yes | Reduced frequency on first attempt, not tolerated on second attempt | None reported | Lethargy, need for nasogastric supplementation on second attempt |
| A36 | 3 years | Daily | At least 6 months | Yes | Initial seizure freedom, later returned to less than baseline | Increased alertness | Vomiting, lethargy |
| A37 | 2 years 1 month | Daily | 9 months | Yes | 2-week seizure freedom, gradual return to baseline | None reported | Food refusal, loss of head control and feeding difficulties which improved upon weaning of diet |
| A39 | 1 year 6 months | Unknown | 6 months | No | No change in seizures | Increased alertness | Feeding difficulties |
| A47 | 10 months | Unknown | At least 4 months | Yes | 2-week seizure freedom, then recurrence | None reported | Constipation |
| A48 | 6 months | Daily | At least 2 months | Yes | Seizure freedom starting 2 weeks after initiation | None reported | None reported |
| A56 | 6 weeks | Daily | At least 8 months | Unknown | Unquantified improvement | None reported | None reported |
| A64 | 3 months | 4/day | Unknown | Yes | None | None reported | None reported |
| A73 | Before 2 years, at 4 years | Unknown | Unknown | Unknown | No improvement on first attempt, unquantified improvement on second attempt | None reported | None reported |
| A78 | 8 months | Unknown | At least 6 months | Unknown | None | Unknown | Unknown |
| A79 | 3 years, 5 years, 7 years | Unknown | Unknown | Unknown | None | None reported | Diet not tolerated |
| A81 | 2 years 3 months | Unknown | At least 9 months | Yes | 9 day seizure freedom, then recurrence to below baseline | None reported | None reported |
| A90 | 13 months | Unknown | At least 7 months | Yes | Near resolution for 5 months, later recurrence | Unknown | None reported |
| B1 | 2 years | Daily | 1 year | No | No change in seizures | Yes (unspecified) | None reported |
| B2 | Unknown | Daily | Unknown | No | No change in seizures | Unknown | None reported |
| B3 | 5 years | Daily | 3 years | No | 30% seizure reduction | Unknown | Unknown |
| B7 | 22 months | Daily | 3 months | No | None | Unknown | None reported |
| B9 | 3 years | Daily | 1 year | No | None | Unknown | None reported |
| B10 | 7 years | Daily | 8 years | No | None | Unknown | None reported |
| B11 | 4 months | Daily | 10 months | No | None | Unknown | None reported |
| B13 | Unknown | Daily | Ongoing | No | None | None reported | None reported |
| B14 | 1 year | Unknown | 4 months | No | None | None reported | Viral gastroenteritis |
| B15 | 17 months | Daily | 20 months | No | None | Unknown | None reported |
| B17 | 3.75 years | Daily | Ongoing | Unknown | Loss of efficacy | Unknown | Unknown |
| B18 | 10 years | Daily | Ongoing (At least 5 years) | Unknown | Lack of efficacy | Unknown | Unknown |
| B19 | 1 year | Unknown | 6 months | No | None | Unknown | None reported |
| B20 | Unknown | Daily | Unknown | Unknown | None | Unknown | None reported |
| B21 | 5 months | 2-3/week | Ongoing | No | None | Unknown | None reported |
| B22 | 2 years | 6-7/day | 6 months | Yes | Reduced seizures to 0-2/day | Unknown | None reported |
| B23 | 3 months | 2/day | Ongoing | Unknown | Seizures better controlled with ketogenic diet and levetiracetam (not quantified) | Unknown | None reported |
| B25 | 11 months | 2-3/day | Ongoing | Yes | Reduced seizures to 0-2/day | Unknown | Excess ketosis, acidosis |
| B26 | 12 months | 8/day | Ongoing | Yes | Reduced seizures | Unknown | None reported |
| B28 | 2.5 years | Daily | 3 months | Unknown | Uncertain benefit | Unknown | None reported |
| B29 | 14 months | Daily (10-30) | 2 years | Yes | Initial response then seizure recurrence | Unknown | GI upset that seemed to trigger seizures |
| B30 | 10 months | Daily | Ongoing | Yes | None | Yes (unspecified) | None reported |
| B31 | 12 months | Daily | 5 months | Unknown | Lack of efficacy | Unknown | None reported |

Supplemental Table 2. Response of 126 individuals with CDD to common sleep and behavioral medications

| **Category** | **Name of medication** | **Indications** | **Number of individuals tried** | **Positive response** | **Worsening symptoms** | **No change** | **Unknown response** |
| --- | --- | --- | --- | --- | --- | --- | --- |
| Sleep | Melatonin | Insomnia | 30 | 10 | 0 | 5 | 15 |
| Sleep | Clonidine | Insomnia | 8 | 2 | 0 | 1 | 5 |
| Sleep | Trazodone | Insomnia | 5 | 1 | 0 | 0 | 4 |
| Sleep | Gabapentin | Insomnia | 4 | 0 | 0 | 1 | 3 |
| Sleep | Clomipramine | Insomnia | 1 | 0 | 0 | 0 | 1 |
| Sleep | Diphenhydramine | Insomnia | 1 | 1 | 0 | 0 | 0 |
| Sleep | Doxepin | Insomnia | 1 | 1 | 0 | 0 | 0 |
| Sleep | Mirtazapine | Insomnia | 1 | 0 | 0 | 1 | 0 |
| Sleep | Ramelteon | Insomnia | 1 | 0 | 0 | 0 | 1 |
| Sleep | Suvorexant | Insomnia | 1 | 0 | 0 | 0 | 1 |
| Sleep | Temazepam | Insomnia | 1 | 0 | 0 | 0 | 1 |
| Behavioral | Clonidine | Irritability | 6 | 2 | 0 | 1 | 3 |
| Behavioral | Risperidone | Guttural “buzzing” sound, OCD-like symptoms, agitation, stereotyped behaviors | 2 | 0 | 0 | 1 | 1 |
| Behavioral | Methylphenidate | Attention deficit, daytime drowsiness | 2 | 1 | 0 | 0 | 1 |
| Behavioral | Clonazepam | Irritability | 2 | 1 | 0 | 0 | 1 |
| Behavioral | Non-FDA-approved cannabis derivative | Vocalizations, shrieking/kicking | 2 | 0 | 0 | 0 | 2 |
| Behavioral | Aripriprazole | Guttural “buzzing” sound, OCD-like symptoms | 1 | 0 | 0 | 1 | 0 |
| Behavioral | Clomipramine | Guttural “buzzing” sound, OCD-like symptoms | 1 | 0 | 0 | 0 | 1 |
| Behavioral | Citalopram | Guttural “buzzing” sound, OCD-like symptoms | 1 | 1 | 0 | 0 | 0 |
| Behavioral | Cyproheptadine | Guttural “buzzing” sound, OCD-like symptoms | 1 | 0 | 0 | 1 | 0 |
| Behavioral | Diazepam | Not specified | 1 | 0 | 0 | 0 | 1 |
| Behavioral | Escitalopram | Guttural “buzzing” sound, OCD-like symptoms | 1 | 0 | 0 | 0 | 1 |
| Behavioral | Lorazepam | Guttural “buzzing” sound, OCD-like symptoms | 1 | 1 | 0 | 0 | 0 |
| Behavioral | Nitrazepam | Irritability | 1 | 0 | 0 | 0 | 1 |
